# Genome-wide linkage scan for loci associated with epilepsy in Belgian shepherd dogs

**DOI:** 10.1186/1471-2156-11-35

**Published:** 2010-05-04

**Authors:** Anita M Oberbauer, Janelle M Belanger, Deborah I Grossman, Kelly R Regan, Thomas R Famula

**Affiliations:** 1 Department of Animal Science, University of California, Davis, CA, USA

## Abstract

**Background:**

Idiopathic epilepsy in the Belgian shepherd dog is known to have a substantial genetic component. The objective of this study was to identify genomic regions associated with the expression of generalized seizures in the Belgian Tervuren and Sheepdog.

**Results:**

DNA from 366 dogs, of which 74 were classified as epileptic, representing two extended families were subjected to a genome-wide linkage scan using 410 microsatellite markers yielding informative coverage averaging 5.95 ± 0.21 Mb. Though previous studies based on pedigree analyses proposed a major gene of influence, the present study demonstrated the trait to be highly polygenic. Studies of complex disorders in humans indicate that a liberal composite evaluation of genetic linkage is needed to identify underlying quantitative trait loci (QTLs). Four chromosomes yielded tentative linkage based upon LOD scores in excess of 1.0. Possible QTLs within these regions were supported also by analyses of multipoint linkage, allele frequency, TDT, and transmission of haplotype blocks.

**Conclusions:**

Taken together the data tentatively indicate six QTLs, three on CFA 2, and one on each of CFA 6, 12, and 37, that support fine mapping for mutations associated with epilepsy in the Belgian shepherd. The study also underscores the complexity of genomic linkage studies for polygenic disorders.

## Background

Belgian Tervuren (BT) and Belgian Sheepdogs (BS) (collectively referred to as Belgian shepherd dogs outside of the United States) have a long history of idiopathic epilepsy [1,2]. An early study found the prevalence of epilepsy for BT in the United States to be 17% [3] which is higher than the estimated prevalence of 9.5 - 11.1% for BT and BS in Denmark [2]. In the general dog population, the prevalence of epilepsy is estimated to be approximately 1.0% [4]. Regardless of the precise prevalence of idiopathic epilepsy, the prevalence in the Belgian shepherd is perceived to be too great and efforts to reduce the incidence are desirable.

As in other breeds [5-8], the presumed mode of inheritance for idiopathic epilepsy in the Belgian shepherd is polygenic with a recessive gene of major influence [3,9]. In addition to a genetic component, other factors are believed to contribute to the expression of epilepsy, possibly by lowering the seizure threshold; these factors include stress [2,10] and sex steroids [2]. In general, seizure activity in the Belgian shepherd begins about 4 years of age [2,3] and treatment varies from none, to anticonvulsive medication (phenobarbital and/or potassium bromide), to euthanasia for intractable cases.

Due to the similarities of seizure activity between humans and dogs [11], the use of the human International League Against Epilepsy (ILAE) scheme [12] has been proposed for the classification of seizures for dogs to improve consistency in data recording [2,11]. Using the ILAE system, Berendt [2] completed a comprehensive survey of epilepsy in the Belgian shepherds of Denmark and reported the prevalence of epilepsy at 9.5% (49 out of 516 dogs). Of these 49 epileptic dogs, 71% of the dogs experienced either primary or secondarily generalized seizures.

Identification of a locus linked to the expression of epilepsy would assist in reducing the prevalence of idiopathic epilepsy in the Belgian shepherd and may also provide clues to mutations contributing to epilepsy in other breeds. Genetic testing currently is used by dog breeders seeking to improve the health of their breeds as evidenced by participation in the Canine Health Information Center [13] while compulsory implementation of existing genetic tests as a condition of registry is being considered in the United Kingdom [14]. Further, the similarities of seizure disorders between humans and dogs [15] suggest that the findings of one species can aid the progress in the other. With that objective, a genome-wide linkage study was undertaken for the Belgian shepherd dog.

## Methods

### Animals

Dog owners who participated in the study were recruited with cooperation from the American Belgian Tervuren Club, the Belgian Sheepdog Club of America, interested breeders, and individual owners. Research protocols were approved by the Institutional Animal Care and Use Committee of University of California, Davis. The present study included purebred Belgian Tervuren (BT) and Belgian Sheepdogs (BS) affected with epilepsy, as well as unaffected dogs. Genomic DNA was obtained by owners submitting three buccal swabs from each dog. Along with the DNA sample, owners provided specific seizure information (frequency, onset and duration of seizures), veterinary reports, and a three-generation pedigree. The dogs were designated as phenotypically epileptic based on information provided by the owner and veterinarian. Dogs were defined as epileptic if they exhibited repeated "epileptic seizure phenomenology" common to generalized seizures based upon the classification system used for humans and applied in dogs [2].

The BT data set consisted of 355 dogs, including 177 dogs for which buccal swab derived DNA was available for analysis and 178 additional dogs included to construct the appropriate pedigrees for analysis (Additional File 1). Of the dogs for which DNA was available, 134 were designated as unaffected, 36 designated as having affected epilepsy status, and 7 having indeterminate ("unknown") status. The dogs without DNA submitted and included to complete the pedigrees were largely designated as "unknown" for the status except for deceased dogs known to meet the epileptic designation criteria (five dogs). Dogs classified as epileptic were equally represented by males and females (18 and 18, respectively; p > 0.5). For the dogs classified as epileptic, the average age at first observed seizure was 47.5 months, an age comparable to previously published data [2,3]. Dogs were designated as "unaffected" if they had no reported seizure activity and were over 6 years of age; six years of age was selected because nearly 90% of BT and BS dogs defined as epileptic were under the age of 6 years at age at first recorded seizure (Additional File 2).

The BS breed, which is highly related to the BT [16], was used to independently assess the findings of the BT association. The BS data set also consisted of 355 dogs, including 189 dogs for which buccal swab derived DNA was available for analysis and 166 additional dogs (again included to construct the appropriate pedigrees for analysis with epilepsy status classified as unknown). Of the dogs for which DNA was available, 147 were designated as unaffected, 38 designated as having affected epilepsy status, and 4 were classified as unknown. Similar to the BT male and female distribution, epileptic BS dogs were equally distributed between the two sexes (p > 0.5). The average age at seizure onset in the BS dogs was 45.5 months.

### DNA collection, microsatellite markers and PCR methods

Genomic DNA was extracted from the buccal swabs [17] and used in a whole genome-wide linkage study. An initial genome scan of 100 microsatellite markers was run on a modest number of BT [9] and expanded in the present study for a total of 436 microsatellite markers. Markers were selected from various sources, including the 1999 canine genetic linkage map [18], Minimal Screening Set 2 [19] and the University of California, Davis linkage map [20]. Criteria used in marker selection were to provide 9-Mb coverage of the canine genome and the ability to be amplified by PCR in multiplex reactions. Microsatellite markers were amplified and resolved in multiplexed sets using standard procedures [21]. A high-throughput scanning fluorescence detector system was used to resolve the labeled PCR products (Applied Biosystems, Life Technologies, Carlsbad, CA) and genotypes were captured by STRand genotyping software v2.3.106 (STRand, Davis, CA available at http://www.vgl.ucdavis.edu/STRand) [22].

### Statistical analyses

Several linkage analyses were undertaken; the first making use of the variance components strategy introduced by Almasy and Blangero [23]. Two-point and multipoint linkage analyses were performed using the SOLAR software package v4.2.0. Two phenotypes were considered in these analyses, one the binary trait of epilepsy (using a liability threshold model as outlined in Duggirala et al. [24], the second trait was age at onset for this seizure disorder. Age at onset was evaluated as a continuous variable, taken as the Martingale residual from the Cox proportional hazards model fit on age of first seizure, accommodating censoring, as suggested by Yoo et al. [25]. The proportional hazards model took the following algebraic form(1)

where y_ij _is the observed age at first seizure (including accommodation for censoring in those animals that are free of disease), μ is an unknown constant, sex is the effect of the *i*-th sex (*i *= 1,2) and e_ij _is an unknown residual. Computation of the Cox proportional hazards model and the ensuing Martingale residuals was facilitated through the survival package of the public domain language R (version 2.9.1) [26].

Epilepsy was evaluated with or without correction for age at onset as well as sex of dog. Algebraically, this yields a model of(2)

where y_ij _is the observed epilepsy phenotype (yes/no), μ is an unknown constant, sex_i _is the effect of the *i*-th sex (*i *= 1,2), β is the regression coefficient for age at first seizure and e_ij _is an unknown residual. When age at onset was evaluated as a dependent variable, the effect of the sex of dog was included as an independent variable. For either phenotype, a two-point analysis was done, moving to the multipoint analysis for chromosomes suggested to play an important role in the expression of phenotype.

An additional two-point linkage analysis was undertaken with CRI-MAP v2.4 according to published instructions [27]. Application of this technique requires an assumption about the potential for a single locus contribution to the expression of epilepsy, along with a suggested mode of inheritance (i.e., recessivity/dominance). Though previous analyses of pedigrees suggested a major locus of large effect impacting epilepsy in the Belgian shepherd dog [9], the variance component strategy of Almasy and Blangero [23] does not require assumptions on the mode of inheritance and genetic parameters for putative QTLs.

For the chromosomes of interest, the simple technique, sib-pair analysis, proposed some time ago by Haseman and Elston [28] was used. The technique regresses the square of phenotypic differences between sibs on the identity by descent values for marker genotypes in the two sibs [29]. The identity by descent values were those computed with SOLAR v4.2.0, and regressions computed with the public domain language R [26].

Relationships between disease status and the presence or absence of various marker alleles (or allelic combinations) were evaluated by the Fisher exact test using published criteria for linkage between the marker and a genetic locus for epilepsy [30] though this test is more suited for random samples than samples including familial relationships. Sensitivity and specificity analyses were performed to assess the diagnostic value of the presence/absence of disease with a given genotypic classification [31].

Haplotypes were generated with Mendel 8.0 (Haplotype option 3) [32]. The magnitude of the pedigree required the BT and BS families to be split into smaller family structures composed of 20-50 dogs focused around founding sires with affected and unaffected offspring. Affected dogs and their parents were analyzed by the transmission/disequilibrium test (TDT) using Mendel 8.0 (TDT Analysis Option 13) [32]. The TDT analysis assigns P-values by using a permutation procedure that shuffles alleles as passed or not passed to each offspring from its parents. Within the dataset there were three dogs in which both parents were genotyped, 18 dogs with one parent genotyped and one parent lacking genotypic information, and 14 dogs for which both parents had unknown genotype. For parental dogs in which DNA and genotypes were not available, haplotype alleles were assigned by the MENDEL haplotype option by using the genotypes of offspring or, if only a single offspring, the allele frequencies within the family with the highest frequency allele assigned. Haplotype blocks composed of four markers surrounding markers with suggestive two-point linkage LOD scores were determined. After identifying each unique haplotype, haplotype frequencies within a block for each phenotype were compared by χ2 statistics. Though multiple comparisons were made, and the results need caution in interpretation, assessing haplotype frequencies were merely one in a number of linkage assessments. Further, following the recommendations of Rothman, as well as Perneger [33,34], adjustments for multiple comparisons were not performed.

## Results

### Genome-wide markers

Of the 436 microsatellite markers used, 410 consistently amplified the genomic DNA (Additional File 3) and had verifiable chromosomal positions on the complete canine genome map [35,36]. Of those, 353 yielded PIC values equal to or in excess of 0.30 in the two breeds (informative markers) with a high of 0.9 (representing 16 distinct alleles); the mean and median PIC value was 0.58 for markers with PIC values ≥ 0.3 or above. An additional 37 markers gave PIC values between 0.10 and 0.30. The informative markers provided an average coverage of 5.95 ± 0.21 Mb and a median coverage of 5.41; the largest block without a PIC > 0.3 marker coverage was 32.36 (one block) and 11 regions >15 Mb (including the 32.36 block). Linkage between the informative markers was validated using the two-point option in CRI-MAP.

The BT and BS pedigrees used in the present study were modeled empirically to assess if they had the power to generate chromosome-wide significant linkage between the epileptic phenotype and the markers used. Using simulated marker genotypes, the simulations demonstrated that the families composed provided sufficient power to detect linkage with the SOLAR analyses. LOD scores in excess of 12.0 were possible for these pedigrees if such linkage existed and the disorder was inherited as an autosomal recessive with a single gene of major influence as previously predicted [3]. However, the complexity of the expression and characterization of the disorder along with the polygenic nature, would suggest LOD scores of lower magnitude to be expected.

### Linkage

Applying standards used in genome-wide linkage studies of human polygenic disorders [37-39], SOLAR LOD scores in excess of 1.0 were considered suggestive of linkage. Four different chromosomes had regions that met the LOD threshold criterion. Table 1 presents the two-point LOD scores from the binary trait of epilepsy and the continuous trait of age at onset analyses. The LOD scores generated from the multipoint analyses for the binary trait of epilepsy are presented as the highest multipoint LOD score nearest the associated marker. The dichotomous nature of the age at onset observed in the present data suggests that adjustment for age at onset was not necessary, a finding supported by the marginal improvement in two-point LOD scores when age at onset was evaluated. The tentative QTLs were located on chromosomes CFA 2, 6, 12, and 37. For all markers examined the strongest two-point BT LOD scores for the binary trait of epilepsy were found on CFA 12: 1.221 and 1.212 for FH3591 and FH2054, respectively. SOLAR LOD scores for the BS supported the findings in the BT. For the BS dogs, the strongest BS LOD scores for all markers examined were found on CFA 37: 1.382 and 1.159, for AHT133 and REN67C18 respectively; these markers corresponded to the same region as the highest BT CFA 37 multipoint LOD score. Significance for the BT and BS dogs was also detected for these four chromosomal regions by the Fisher exact test (Table 1).

**Table 1:** Association of microsatellite markers with epilepsy in Belgian Tervuren dogs on chromosomal regions exhibiting suggestive linkage

CFA Position Mb	Marker	PIC	Two-Point Epilepsy^a^	Two-Point Age at Onset^b^	Multi-point LOD^c^	Fisher Exact*	Cri-Map LOD	TDT*	Sib-Pair*	Hap block*
**CFA02**										
7.16	FH2274	0.41	0.615	0.715	0.63	0.0018	0.02	0.002	0.175	
10.35	v170	0.57	0.396	0.781	0.785	0.0001	0	0.000	0.333	0.04
15.5	v163	0.55	1.095	1.616	0.802	0.0005	0	0.000	0.413	
43.23	v200	0.65	0.037	0.129	0.074	0.0067	0	0.388	0.142	
46.62	v205	0.56	1.063	1.447	0.08	0.3283	0.24	0.005	0.144	
69.34	v235	0.63	0.252	0.133	0.864	0.0104	0.24	0.002	0.56	
73.02	v240	0.69	1.001	1.415	0.834	0.0076	0.84	0.001	0.097	0.05
78.35	AHT111	0.59	0.01	0.068	0.733	0.0003	0.38	0.009	0.542	
81.01	FH3965	0.78	0.15	0.031	0.735	0.7088	1.13	0.363	0.102	
**CFA06**										
15.58	v620	0.41	1.057	1.024	0.236	0.6055	0.15	0.786	0.12	0.008
26.16	v633	0.34	0.251	0.362	0.015	0.6572	0	0.876	0.447	
30.97	v639	0.70	0.073	0.161	0.004	0.0283	0.02	0.000	0.454	
**CFA12**										
24.28	1-21-3	0.68	0.719	0.79	0.625	0.114	0.31	0.000	0.999	
24.28	FH2054	0.67	1.212	1.26	0.634	0.5973	0.83	0.010	0.464	
27.72	FH2401	0.61	0.15	0.448	0.688	0.667	0	0.094	0.844	
35.37	v1172	0.39	0	0.077	0.751	0.6036	0	0.010	0.905	
44.55	FH3591	0.85	1.221	1.369	0.788	0.3102	0.2	0.002	0.929	0.035
**CFA37**										
18.09	AHT133	0.53	0.048	0.066	0.217	0.0021	0	0.013	0.288	0.003
22.39	Ren67c18	0.59	0.391	0.528	0.960	0.007	0	0.016	0.025	

^a ^Two-point LOD scores for the binary trait of epilepsy

^b ^Two-point LOD scores for the continuous trait of age at onset

^c ^Highest multipoint LOD score for the binary trait of epilepsy nearest the associated marker

*P-values

To corroborate those findings, additional tests of linkage were performed (Table 1) including a second linkage analytical program (CRI-MAP) and sib-pair analysis. Allelic transmission was evaluated by the TDT test and assessing haplotype blocks. Only two CRI-MAP LOD scores exceeded 1.0: at the marker located approximately at 81.01 Mb on CFA 2 and at 55.33 Mb on CFA 12. The CRI-MAP suggestive linkage on CFA 2 was within the region identified by the two-point analyses whereas the CFA 12 suggestive linkage was adjacent to the region identified. Sib-pair analyses were only significant for CFA 12 (in a region adjacent to, but not within, the region identified by the two-point) and CFA 37 in the BT. For the BS, only CFA 9 had significant sib-pair linkage; CFA 9 did have suggestive linkage in both the BT and BS based on the Fisher and TDT assessments but the LOD score was not supportive. The TDT and haplotype block tests agreed with the association identified by the two-point linkage analyses. For the BT dogs, CFA 2, 6, 12, and 37 showed specific alleles (p < 0.05) being passed more to affected dogs than to unaffected dogs suggesting that the markers on these chromosomes are disease-predisposing or closely linked to such a locus.

Taken together, there were six genomic regions exhibiting tentative QTLs for the epileptic phenotype; one chromosome, CFA 2, had more than one region associated. The approximate locations of QTLs were CFA 2 at 7.16-15.50 Mb, 43.23 - 46.62 Mb, and 69.34 - 81.01 Mb; CFA 6 at 15.58-30.97 Mb; CFA 12 at 24.28 - 44.55 Mb; and CFA 37 at 18.09 - 22.39 Mb.

## Discussion

The aim of this work was to detect microsatellite markers associated with the epileptic phenotype in the Belgian shepherd. Using multiple assessments, we sought congruence of the results to identify linkage to specific genomic regions. Given that the disorder is complex in expression and in inheritance, LOD scores achieving classical significance in excess of 3.0 were not achieved. Lower LOD scores that are viewed as suggestive of linkage are frequently used in genome linkage studies for human polygenic traits and disorders [37-39] as well as complex disease in horses [40]. The complex nature of epilepsy in the dog would also suggest a liberal interpretation of potential linkage indicators is appropriate. By applying numerous assessments to confirm suggestive LOD scores, the likelihood of identifying false linkage was reduced.

Within the population studied, males and females were equally affected and the age at onset was, for the vast majority of dogs, prior to 6 years of age. Dogs in which the first seizure was reported at older ages may reflect a different form of epilepsy representing different mutations, or quite possibly earlier seizure episodes that went unnoticed by owners. The dataset demonstrated that although the risk of expressing epilepsy changes with age, the risk of expressing epilepsy drops precipitously with increasing age. Specifically, epilepsy, like many disorders would be expected to have a significant age at onset component. Any linkage analysis should therefore accommodate this anticipated effect. Yet as the counts in the supplemental table reveal, there was a dichotomous nature to age at onset in the present data suggesting age at onset was accounted for by the trait of epilepsy. Indeed, the polyserial correlation of age of observation and epilepsy status was -1.0 (computed with package polycor in R) [26]. The linkage analysis for age of onset measured as the Martingale residual yielded results nearly identical to the binary trait of epilepsy without any age adjustment, as should be expected if age of observation and epilepsy status were so closely related.

In some cases single LOD scores were potentially interesting but unsupported by adjacent markers, multipoint, or Fisher exact test. Based upon multiple assessments of linkage and association, six genomic regions on four chromosomes were found to tentatively harbor QTL associated with epilepsy in the Belgian shepherd. The linkage blocks, although large, perhaps represent the best available evaluation using a linkage association based on microsatellites and their incumbent limitations. For example, 13% of the microsatellites specifically chosen for their allele diversity were uninformative for use in the BT and BS populations. In contrast, microsatellites seem to be very useful in the exclusion of potential candidate genes [41]. If applied to the present study, numerous chromosomes can be excluded from further consideration as harboring major QTL associated with epilepsy.

The rather limited utility of the sib-pair analysis was not unexpected given the design of the experiment as a familial linkage study and the incomplete availability of littermates from a given breeding. However, all the data taken together do provide compelling evidence of suggestive QTLs in influencing the expression of epilepsy in the Belgian shepherd dog. From the present study microsatellites appear to lack resolution to clearly identify possible causal mutations for a complex, variable phenotypic disorder. Fine mapping of these regions will require greater saturation of markers to generate greater haplotype diversity using the genetic structure of the dog [42].

There are numerous positional candidate genes, based upon their involvement in human epilepsy, possible for these six regions. Syntenic to CFA 2 are the human genes BAFME2 (Benign adult familial myoclonic epilepsy 2) [43] and EIG5 (Epilepsy, idiopathic generalized, susceptibility to, 5) [44]. Candidate genes on CFA 6 based on human studies would be KCTD7, a potassium channel associated with progressive myoclonic epilepsy [45] and EIM [46]. There are 51 identified genes or regions associated with human epilepsy on human chromosome 6 which represents a great deal of synteny with CFA 12; many of these genes/regions are syntenic to the QTL suggested in the present study. A recent characterization of CLN8, a gene involved in human progressive epilepsy and found on CFA 37, was found to be mutated in English Setter dogs having heritable neurodegenerative disease [47]. While CLN8 is not a good candidate gene for the phenotype of the idiopathic epilepsy seen in the Belgian breeds, it does underscore the generalizability of the human and canine gene discovery as it relates to epilepsy. However, the plethora of epilepsy associated genes identified in human families, indicates that caution needs to be applied when investigating candidate genes and that a more precise localization would be a more prudent approach for polygenic disorders.

The absence of robust association may be due to the polygenic nature of the disease as noted before. Further aspects that confound the study of epilepsy are the accuracy of seizure characterization, the subtle differences in seizure presentation that while appearing to be common in terms of expression represent differences in underlying genetic causality, and the classification as idiopathic epilepsy in the absence of a positive diagnosis. Recent reports of canine epilepsy suggest that the genetics involved in seizures may be distinctly different within the different lines of a single breed [11]. Additionally, some disorders described as polygenic are the result of copy number variations [48] and the approach of linkage mapping used in the present study would not identify copy number variability. Another consideration in complex diseases is the concept of "common disease/common variant" hypothesis whereby it is assumed that a common complex disease is caused by few but common genetic variants. However, it is also likely that a particular combination of relatively rare alleles could also evoke the disease [49]. If the latter is the case for canine epilepsy then the classical linkage studies may be insufficient to detect the genomic regions.

## Conclusion

The composite data presented here suggests six chromosomal regions on four chromosomes may tentatively harbor QTLs involved in generalized idiopathic epilepsy in the Belgian shepherd dog. Fine mapping is necessary using single nucleotide polymorphisms which should enable the identification of genes and mutations in predisposing a dog to seizure activity.

## Authors' contributions

AMO conceived the study, study design, coordination, and drafted the manuscript. JMB participated in coordination, carrying out methods including choosing microsatellites, constructing pedigrees, genotyping, manuscript revision and statistical analyses. DIG participated in carrying out methods including choosing microsatellites, constructing pedigrees, and genotyping. KRR participated in carrying out methods including choosing microsatellites, constructing pedigrees, genotyping and statistical analyses. TRF participated in conceiving the study, study design and statistical analyses. All authors read and approved the final manuscript.

## Supplementary Material

Additional file 1 **Subset of Belgian Tervuren pedigree used in linkage analysis**. An example of the dogs used in the genome-wide linkage analysis. Filled in symbols reflect dogs classified as epileptic, open symbols reflect dogs known to be unaffected, and symbols labeled with a "?" reflect dogs classified as having an unknown status. Click here for file

Additional file 2 **Age Distribution for age at onset of Epilepsy in the Belgian Tervuren and Belgian Sheepdogs**. The data present the characteristic ages of epilepsy diagnosis. Click here for file

Additional file 3 **Genome-wide association of 410 microsatellite markers with epilepsy in Belgian Tervuren dogs**. This table lists the details of all markers used in the microsatellite linkage evaluation. Click here for file
