# Supplementary figures and images for: Genome-wide linkage scan for loci associated with epilepsy in Belgian shepherd dogs
Source: BMC Genet. 2010 May 4;11:35. doi: 10.1186/1471-2156-11-35 (PMC2877138; doi:10.1186/1471-2156-11-35)

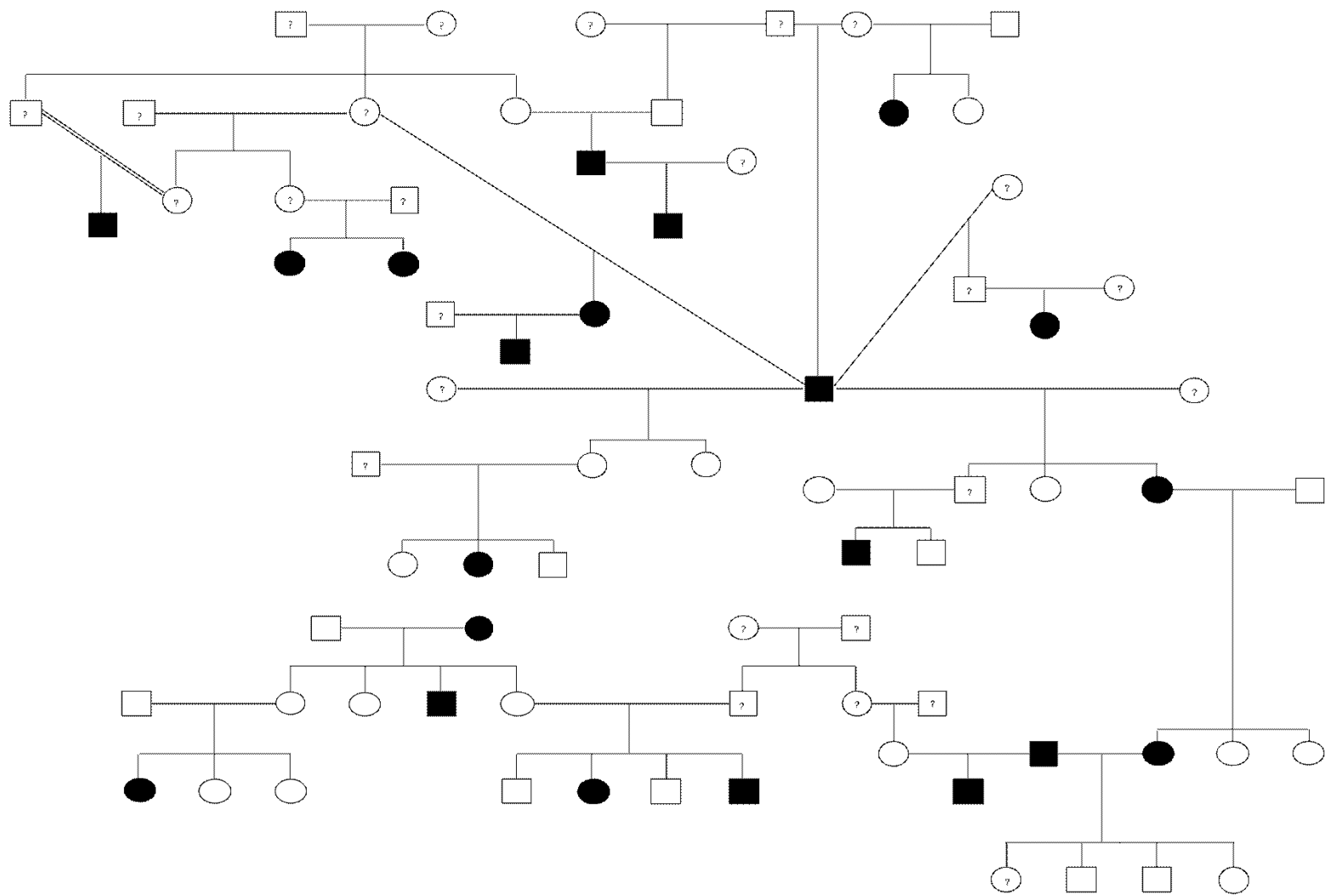

Supplement: Additional file 1 — Subset of Belgian Tervuren pedigree used in linkage analysis. An example of the dogs used in the genome-wide linkage analysis. Filled in symbols reflect dogs classified as epileptic, open symbols reflect dogs known to be unaffected, and symbols labeled with a "?" reflect dogs classified as having an unknown status. [file 1471-2156-11-35-S1.PDF]
